# Early-Stage Investigation of Potential Phosphorus Recovery Pathway from Dredged Sediments: Case of Malmfjärden Bay, Kalmar

**DOI:** 10.3390/molecules31122054

**Published:** 2026-06-11

**Authors:** Rumbidzai Mugwira, Laura Ferrans, Subashini Gnanasekar, Leteng Lin

**Affiliations:** 1 Department of Built Environment and Energy Technology, Linnaeus University, Universitetsplatsen 1, 35252 Vaxjo, Sweden; subashini.gnanasekar@lnu.se (S.G.); leteng.lin@lnu.se (L.L.); 2 Department of Biology and Environmental Science, Linnaeus University, Norrakajplan 6, 39231 Kalmar, Sweden; laura.ferrans@sweco.se

**Keywords:** dredged sediments, phosphorus extraction, metal contamination, circular economy

## Abstract

Metal and nutrients can be extracted and recovered from sediments for beneficial use. The aim of the current study is to extract phosphorus (P) from dredged sediments from Malmfjärden bay through chemical extraction, targeting the determination of the best conditions to extract P from the studied sediments with minimal contamination from metals. The chemicals used for the extractions include citric acid (C_6_H_8_O_7_), sulphuric acid (H_2_SO_4_), sodium hydroxide (NaOH) and ethylenediaminetetraacetic acid (EDTA) at 0.01 M or 0.1 M. The acids were at pH 5, and NaOH at pH 12. The pH was also varied for 0.01 M EDTA at pH 2, and 0.1 M H_2_SO_4_ at pH 1. A single extraction was carried out with each reagent at the varying concentrations and pH. One test had a sequential two-step extraction of EDTA as the first step, followed by H_2_SO_4_ as the second step. The elements were analysed using inductively coupled plasma sector field mass spectrometry (ICP-SFMS). The results showed that the extraction efficiencies were significantly higher at 0.1 M than 0.01 M of the reagents, as well as at more acidic conditions for the reagents with a varied pH, in most cases. H_2_SO_4_ at pH 1 extracted the most P (46.4%), together with most of the other elements. EDTA also exhibited a high extraction efficiency for the elements and can be used as a pre-step to remove metal before extraction with H_2_SO_4_. Alternatively, although it had lower P extraction efficiencies, NaOH can be used as the extractant, since it extracted less of the other elements. The P/metal extraction ratios further indicated that NaOH achieved high selectivity, as well as the second step sequential extraction H_2_SO_4._ Hence, the recovered P will be less contaminated. P extraction from sediments can indeed become one way of recovering the finite element, which would otherwise be normally lost as waste with the sediments.

## 1. Introduction

Marine sediments can harbour various kinds of pollutants, such as metals, persistent organic pollutants and plastics [1,2]. Sediments can also contain high levels of nutrients. The nutrient-rich sediments coming from dredging activities can be useful in the agricultural sector. However, direct use of dredged material might be a challenge as the nutrients in the sediments might not be in an available form for plant uptake [3]. Pollutants in the sediments, such as metals and pathogens, can also be spread during use. The sediments might also not be readily acceptable by end-users, as they are traditionally considered waste. The sediments can thus be amended first, for example, by adding biochar and co-composting [3,4]. Alternatively, the nutrients can be extracted and recovered from the sediments for beneficial use. Phosphorus is one finite resource that can be extracted from the sediments and used as a fertiliser or for other industrial purposes [5].

Sediments act as phosphorus sinks in water bodies. Phosphorus in marine sediments is mainly from sinking particulate matter in the water column or in coastal settings delivered from land by currents [6,7,8,9]. Of the total phosphorus extracted from mines, more than 66% is lost during crop cultivation and livestock production, and 19% through household food waste, mining waste, and fertiliser manufacturing waste [5]. The phosphorus is used and reused in terrestrial ecosystem local cycles approximately 46 times, and finds its way to the ocean, where it is recycled about 800 times by marine organisms before getting into sediments [10].

Phosphorus is mainly attained from mined rock phosphate [11,12]. It is then processed into valuable P-containing products, mainly phosphoric acid (used for fertiliser production) and elemental/white phosphorus (for the production of phosphoric compounds for industrial purposes) [12]. Phosphate rock reserves are controlled by a few countries, chiefly Morocco, China and the US [11], with Morocco having the largest reserve, accounting for more than two-thirds of the reserves [13]. Many researchers have reiterated that with the increase in population and demand, the resource will not be sufficient to meet demand in the next few centuries. Considering that the resource is non-renewable and finite [14], extracting and recovering phosphorus from the various waste streams can be one way of combating the dwindling of the resource.

There are several studies on phosphorus extraction from both liquids (such as wastewater and urine) and solids (such as sewage sludge, manure and soils) [5,15,16,17,18,19,20]. Several potential P recovery techniques have also been employed, which include biological phosphorus removal, precipitation as struvites, hydroxyapatite or calcium phosphates, thermal treatment and chemical extraction [21]. There are several waste streams rich in nutrients, including phosphorus and nitrogen, such as agricultural and industrial effluents, biosolids, municipal wastewater, and animal manure [5].

Dredged sediments are not an exception waste stream, and the recovery implies two steps: firstly, the extraction of phosphorus from the sediments, and secondly, the recovery of phosphorus from the extract containing the phosphorus. The recovered phosphorus can be used for fertiliser production as well as industrial applications, such as food and livestock food additives [11]. Extraction of elements can be done sequentially or by single extraction. Some single P extractions and particularly sequential extractions are primarily done to chemically characterise P in the soil/sediment matrix and not to extract P for subsequent potential P recovery [22].

Single extractions have also been carried out from soils and waste streams such as municipal wastewater/solid waste, animal manure and sewage sludge. P can then be recovered from the extracted solutions. Several studies have been done on potential P recovery pathways from waste streams [5,16,17,20,23,24,25]. There are several factors that affect the extraction process, including the pH, extraction time, reagent, concentration of extractant and liquid-to-solid ratio, and these parameters must be taken into consideration before carrying out the experiment. Table 1 shows some studies that have been carried out where the total extraction of P has been done from solid waste streams. To the best of our knowledge, although various research studies have been conducted on P sequential extraction from sediments, limited investigations on single extractions have been carried out on dredged sediments; hence, the focus of the current study will be on total extractions.

Generally, the chelating agent EDTA is a commonly used complexing reagent due to its high extraction capacity and is thought to extract metals on exchange sites on both organic and inorganic complexes [26]. Metals are solubilised by chelating agents by complexation [27,28]. Other studies have applied EDTA for the extraction of inorganic phosphorus forms, and then H_2_SO_4_ and NaOH for the extraction of organic forms [29]. Principally, the extraction of metals by acids depends on the dissolution of soil components or discrete metal compounds and ion exchange [27]. Dilute strong acids partially extract dissolved trace elements and exchangeable elements associated with organic matter fractions, carbonates, and Fe or Mn oxides [26]. NaOH also extracts organic phosphorus [29,30]. The efficiency of P potential recovery using NaOH does not usually exceed 40% [31]. However, the recovered product from precipitation experiments for P recovery from alkaline leachate could directly be reused as fertiliser or industrial applications as they have low metal contents, with the exception of As [24].

The Kalmar municipality, Sweden, under the Local Water Conservation Projects—Lokala vattenvårdsprojekt (LOVA): Mudster Eco Dredging project, a follow-up of the LIFESURE project, is carrying out some dredging activities with the aim of using the dredged sediments within the circular economy approach. A previous study on the properties of the same sediments investigated from our study showed that they were mainly comprised of silt (62–79%), then clay (14–20%) and sand (7–17%) along with low organic pollutants, low–medium concentration of metals and medium–high nutrient content and organic matter (11.4–14.5%) [32]. Studies on the speciation of metals in the sediments revealed that low concentrations of metals were related to the exchangeable fraction (Zn (4%), and <1% for Cr, Cu, Ni and Pb), whilst major concentrations of the metals were related to the residual part (Cr (70%), Cu (54%), Ni (49%), Zn (37%)) [33,34]. Previously, studies have been carried out on the usage of the sediments as a plant-growing substrate, which has shown some limitations on the sediments being a substrate on their own [4].

Although phosphorus recovery has been widely studied in conventional waste streams, the recovery potential of dredged marine sediments has received far less systematic attention, particularly in relation to single-step and recovery-oriented extraction approaches that are relevant for practical implementation. Most previous work on sediments has focused on phosphorus fractionation and environmental speciation, rather than on how extraction chemistry can be optimised to recover P while controlling the transfer of metals into the extract. This represents a significant knowledge gap, because the feasibility of the circular reuse of dredged sediments depends not only on how much phosphorus can be mobilised, but also on whether the recovered P can meet acceptable quality in terms of contamination. The present study addresses this gap by evaluating a set of chemically distinct extractants under different concentrations and pH conditions, and by testing a sequential EDTA–H_2_SO_4_ strategy to separate metal removal from phosphorus mobilisation. The study is novel in that it links sediment remediation concerns with phosphorus resource recovery, thereby reframing dredged sediment from a waste requiring management to a potential secondary raw material. This contribution is important for advancing circular sediment use, reducing dependence on finite phosphate rock, and supporting the evidence-based development of recovery pathways for nutrient-rich but potentially contaminated sediments.

## 2. Results and Discussion

### 2.1. Sediment Characterisation

The concentration of P and the other elements in the sediment was determined (see Table 2). The concentration of P was 1080 mg kg^−1^ DW. Previous studies on the sediments from the same bay showed the result of 1300 mg kg^−1^ [4], which can relate to the current study. Some studies on the Baltic sediments have shown P concentrations ranging from 619 mg kg^−1^ to 3973 mg kg^−1^ [35,36,37,38,39,40]. Most of the Baltic Sea has been assessed as eutrophic as a result of surplus nutrients, particularly nitrogen and phosphorus, and action plans are being implemented to reach the goal of a Baltic Sea unaffected by eutrophication [41].

For the trace metals, the concentrations were compared to the Swedish guide values for contaminated soils as stipulated by the Swedish EPA [42]. The guideline values are for *Känslig markanvändning* (KM), which is sensitive land use areas, and for *Mindre känslig markanvändning* (MKM), which is less sensitive land use areas. All the metal concentrations were below the less sensitive land use limit. This concurred with the results of previous studies on sediments from the same bay [32,33,43]. The concentration of Cd, Pb and Zn surpassed the sensitive land use limits, whilst that of As was close to the limit. Similar results were observed with some metal concentrations equal to or above the KM limit for As, Pb and Cd [32,33]. The Zn concentrations were, however, below the KM limit in the study. Similar results as in the current study were also observed for sediments with high nutrients and sediments with polymer in their study [4]. Some studies on the Baltic sediments have also shown some elevated metal concentration ranges of As (5–276 mg kg^−1^), Cd (0.4–5.5 mg kg^−1^), Pb (40.2–150 mg kg^−1^), and Zn (125–40 mg kg^−1^) [44,45,46,47]. As some of the values exceed the background values of these metals, this could be an indication of anthropogenic pollution into the Baltic marine bodies.

As there are no industrial activities around Malmfjärden bay, the source of contamination in the sediments could be runoff from the surroundings, including an old dumpsite a few kilometres away. The sediments are, however, safe for use in less sensitive land use areas but can be remediated first in order to be used in sensitive land uses. These results are also an indication that for potential P recovery, there are chances of contamination with the other elements that are in the sediments, which may limit the potential of direct use. The remediations of the extractants after P recovery should also be considered to curb environmental contamination from the co-dissolved elements.

### 2.2. Phosphorus and Metal Extraction Efficiencies by the Reagents

The extraction behaviour observed in this study is based on the mineralogical composition of the Malmfjärden sediments and the geochemical stability of P and trace metals. Phosphorus occurs mainly in Ca–P, Fe–P and Al–P phases, while metals such as Cd, Zn, Cu and Ni are associated with carbonate and Fe/Mn oxyhydroxide fractions, and Cr and Pb are largely incorporated into residual silicate minerals. The contrasting extraction efficiencies of the reagents can be explained by their distinct geochemical dissolution pathways and interactions with sediment mineral phases. H_2_SO_4_ operates through proton-promoted dissolution, where high H^+^ activity attacks Fe/Mn oxyhydroxides and Ca–P phases, destabilising mineral surfaces and releasing both phosphate and co-precipitated metals such as Cd, Zn, Ni and Cu. This mechanism explains the strong extraction observed at pH 1, where extensive protonation enhances the dissolution of Fe–P, Al–P and Ca–P [48] whereas EDTA and citric acid mobilise metal through ligand-promoted dissolution where chelating agents dissolve metals through complexation, which removes metal ions from mineral surfaces. EDTA forms highly stable complexes with Cd, Cu, Ni, Pb, and Zn ions, resulting in the high extraction of cationic metals but limited release of P unless there are metal–phosphate associations. Citric acid, a weaker organic ligand, partially dissolves Fe/Al-bound P and mobilises metals through complexation with Fe and Al ions, consistent with its intermediate extractionbehaviour [49]. Under alkali conditions, NaOH follows a hydroxide-promoted desorption. At high pH, Fe/Al oxide surfaces become negatively charged (above their point of zero charge), causing the electrostatic repulsion and desorption of P and As oxyanions. This explains the selective extraction of P and As in NaOH. Meanwhile, cationic metals Cd, Zn and Cu form insoluble metal hydroxides, which either precipitate or remain strongly bound to the solid phase, resulting in minimal metal extraction. These behaviours reflect the fundamental differences between oxyanion mobility and cationic metal sorption under varying pH conditions [50]. Together, these mechanistic pathways clarify why acidic conditions favour the co-dissolution of P and metals. They also support the sequential EDTA + H_2_SO_4_ extraction strategy, where EDTA first removes cationic metals and Ca/Mg, followed by the targeted dissolution of P-holding phases by H_2_SO_4_ with reduced co-dissolution of contaminants.

**Figure 1:**
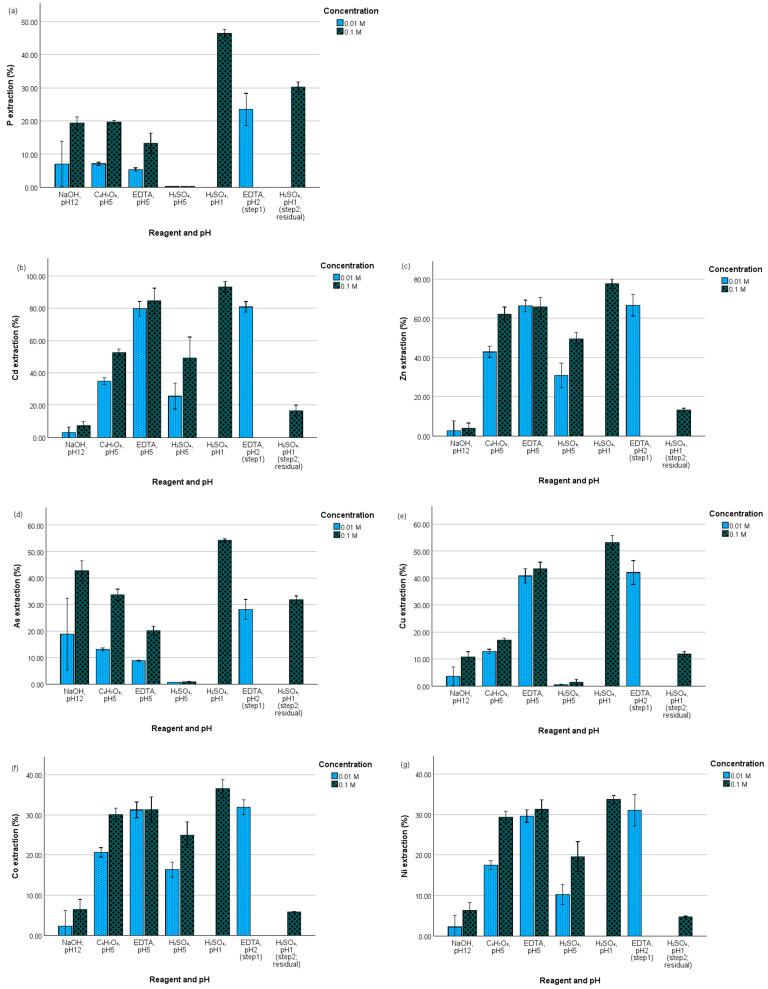

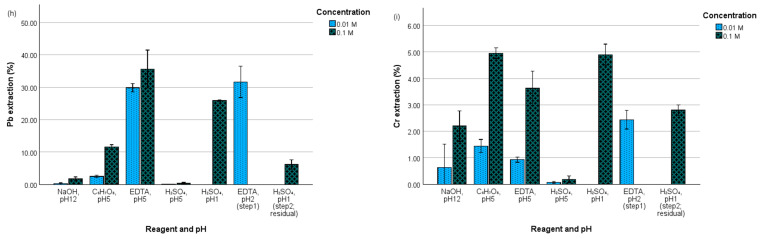
Mean extraction efficiencies for (**a**) P and the metals (**b**) Cd; (**c**) Zn; (**d**) As; (**e**) Cu; (**f**) Co; (**g**) Ni; (**h**) Pb; (**i**) Cr, from Malmfjärden sediments. “H_2_SO_4_, residual” is the second step for the sequential two-step extraction experiment; after pre-extraction with EDTA, pH 2, and then H_2_SO_4_, pH 1, was used for element extraction on the residual sediment. Error Bars: +/− 2 SD.

The results of the element extraction efficiencies by the different reagents at 0.01 M or 0.1 M and selected pH are shown in Figure 1.

Some extraction occurred for all the elements in all the extraction procedures employed, ranging from 0.07 to 93.1% (Figure 1a–i). Generally, the reagents had a higher extraction rate of the elements at 0.1 M compared to 0.01 M. The extraction procedure resulting in the highest extraction rate of P and most metals was 0.1 M sulphuric acid, pH 1. At pH 5, citric acid had the best extraction efficiency of P, followed by EDTA and H_2_SO_4_ had the lowest extraction (Figure 1a). The extraction rates for P were comparable to a previous study on soils under similar conditions, where P was extracted from soil with EDTA, citric acid and water; most P was extracted with EDTA (7.5%), followed by citric acid (5.8%) [16]. In another study on pig manure, citric acid extracted 50–90% P [15]. Another study on sludge ash resulted in NaOH extracting 12–29% P at various concentrations, L/S ratios and extraction times [17]. Extraction with H_2_SO_4_ resulted in rates between 3 and 100% [19]. Variations in extracting conditions, together with P concentrations in the material, can be one reason for variations in the extraction efficiencies of the reagents. Sludge ash P concentrations are usually higher than in sediments, as observed by 69.9–99.0 g kg^−1^ [19] and municipal solid waste incineration fly ash 5.9 g kg^−1^ [20]. Another key factor would be P speciation in the material, which can be done in future studies on Malmfjärden sediments to better understand the P forms in the sediments and the extraction potentials.

Overall, the highest extraction rates were for Cd and Zn (Figure 1b,c), whilst the lowest extraction rates were for Pb and Cr (Figure 1h,i). Cr had the lowest extraction efficiencies of not more than 5% across all the reagents. Cd and Zn also presented the highest extraction for all the reagents except NaOH. Pb and Cr had the lowest extractions for all the reagents except EDTA. For NaOH, As, P and Cu were extracted the most, whilst P and Cr were the least extracted for EDTA. At pH 5, EDTA had the highest extraction rates for most of the metals (Cd, Co, Cu, Ni, Pb, Zn), with Cd extraction rates above 80% (Figure 1b–g). The reagents with the lowest extraction efficiencies of all the elements were NaOH and/or H_2_SO_4_ at pH 5. From a previous study on metal extraction from Malmfjärden sediments [43], the extraction efficiencies using EDTA followed the order of Pb (74.1%) > Zn (66.4%) > Cu (54.3%) > As (47.1%) > Ni (29.7%) > Cr (3.1%). In the current study, for the same elements, the order was Zn (66.3%) > Cu (42.1%) > Pb (32.4%) > Ni (30.6%) > As (19.1%) > Cr (2.3%). Although the order of extraction has some variations, Cr has the lowest extraction rate in both studies. The extraction rates were also close for Zn (both 66%), Ni (30% vs. 31%) and Cr (3% vs. 2%).

From a previous study on the speciation of elements from Malmfjärden sediments, the mobility of metals from the exchangeable/extractable fraction was in the order of Cd > Zn > Co > As > Ni > Pb > Cu. Cr had a high residual fraction, an indication that it is strongly bound to primary and secondary minerals in the sediments [33]. These results are in agreement with the current study, where the overall extraction was in the order of Cd > Zn > As > Cu > Co > Ni > P > Pb > Cr. From another study on the sediments, except for Pb, all the other elements under study (Cu, Cr, Ni, Zn, Fe) had their highest percentage in the residual fraction, with Cr having the highest at 70% [43]. The fractionation is in agreement with the current study, where Cr had the lowest extraction rates. This is probably because the stable Cr(III) in sediments is generally favoured since concentrations of reducing agents commonly surpass the concentrations of the oxidising agents in the sediments [51]. For Pb, as sediments are not homogeneous, the Pb in the current study might have been linked to the residual fraction as well, hence the lower extraction efficiencies.

#### 2.2.1. Effects of Reagents

**Figure A1:**
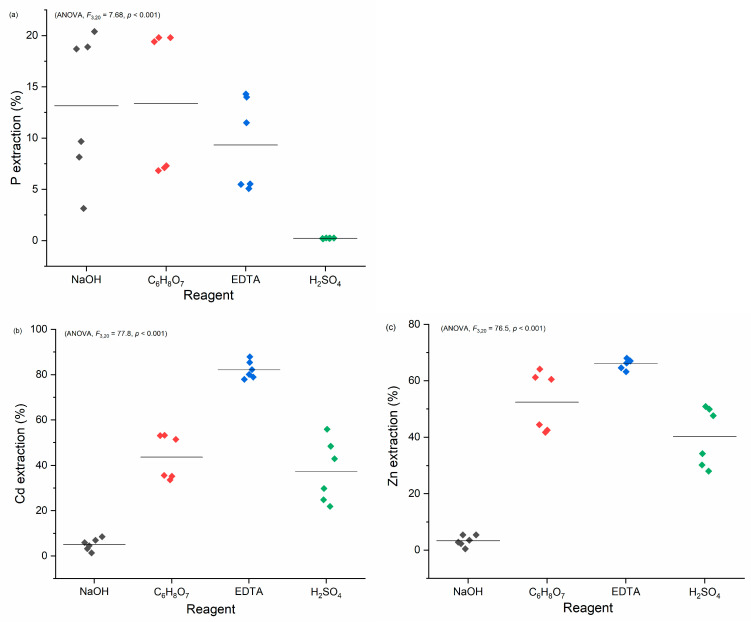

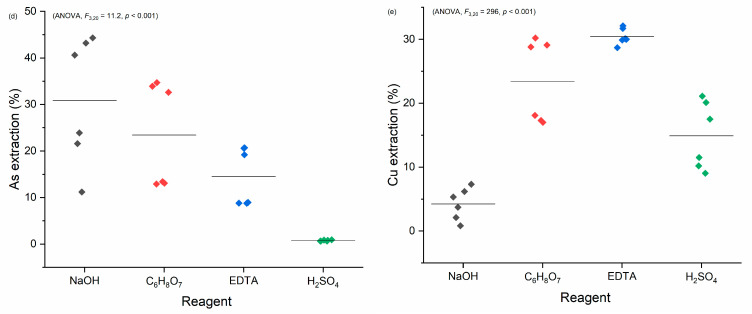

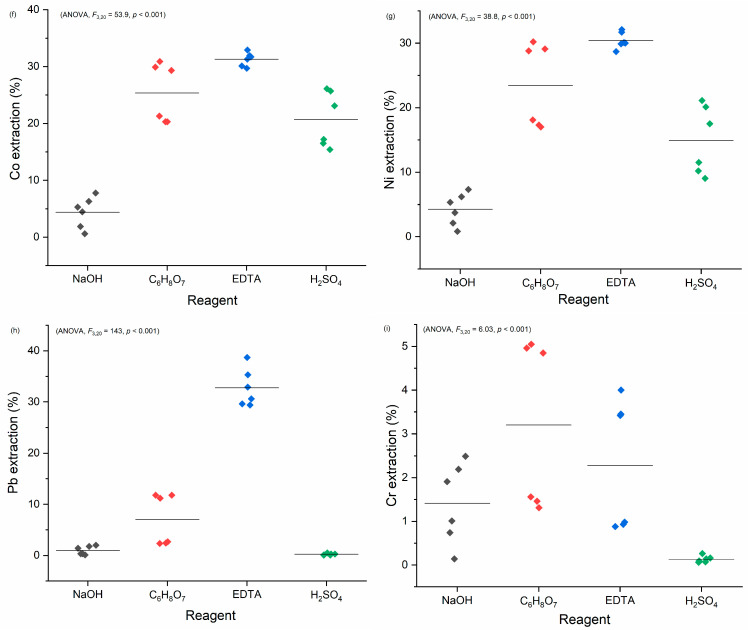
Impact of the type of reagent (NaOH, C_6_H_8_O_7_, EDTA and H_2_SO_4_) on the extraction of all the elements ((**a**) P and the metals (**b**) Cd; (**c**) Zn; (**d**) As; (**e**) Cu; (**f**) Co; (**g**) Ni; (**h**) Pb; (**i**) Cr), with the lines representing the mean extraction.

There was a significant difference in the extraction rates of the reagents for P and all the metals, with the ANOVA test results for all the elements’ extraction being *p* < 0.001. Further results of the overall impacts of the reagent used on the overall extraction of all the elements are shown in Appendix A Figure A1.

EDTA had the highest extraction efficiencies for Cd, Co, Cu, Pb, Ni and Zn. The higher extraction rates for EDTA can be explained by it being a chelating agent with a high affinity for metals, although not for P. However, the partial dissolution of P observed in the study can be due to metal dissolution and the subsequent destabilisation of metal–phosphate bonds [18]. Also, by the complexation of calcium and magnesium, EDTA can dissolve apatite minerals and calcareous material [26], thus liberating the P. For our study, this renders EDTA less favourable as an extractant as it has a higher metal concentration in the solution in proportion to P. There is a higher chance of having higher costs of cleaning off the metals during the subsequent P recovery stage, targeting a smaller fraction of P for recovery. This, however, makes EDTA ideal as a preliminary step to remove a higher percentage of the metal contaminants, with less P extraction.

Citric acid had the highest extraction efficiencies for P and Cr, and the second highest extraction efficiencies for the rest of the elements. This can be as a result of it also being a chelating agent, although weaker. Organic acids, such as citric acid, can enhance P release from Ca, Fe, and Al phosphate minerals in the soil by bonding with Ca, Fe, and Al [52,53,54], although less readily with Ca. Organic acids leach more trace elements, especially Cu, Zn, Pb, and As [18]. In the current study, both trace and major elements were extracted more (Zn, Ca, Cd, Co, As and Ni) with efficiencies of 23–52%. As an organic acid, citric acid is more environmentally friendly compared to the rest of the reagents used in the study, thus having potential as an extractant. However, there is a similar concern as above for EDTA, where there is also a higher metal concentration in the solution, making it an environmental concern for the washing process. Being organic, it can be a cleaner potential reagent for the second step after EDTA cleaning, instead of H_2_SO_4_.

Citric acid and H_2_SO_4_ are both efficacious in dissolving metal oxides and hydroxides. Inorganic acids usually have a higher P extraction efficiency, which can be above 90% [31]. This was observed in the current study, where the highest P extraction efficiency was H_2_SO_4_. The same reagent, however, also had the lowest P extraction efficiency for the whole study at pH 5, where citric acid performed better than H_2_SO_4_ (13% vs. 0.2%), respectively. As stated [55], it is apparent that the extraction efficiency of H_2_SO_4_ is dependent on several parameters, which would include the concentration and temperature, among others. H_2_SO_4_ is a strong acid that thrives better in more acidic conditions. NaOH had the highest extraction efficiencies for As and the second highest for P. P and As exist as oxyanions at high pH, which are solubilised under alkaline conditions. Most trace metals mainly form insoluble hydroxides, which are either precipitated or remain in the substrate [24]. This renders NaOH a cleaner potential P extractant, as P product purification will be minimal compared to the rest of the other reagents.

To enable a direct comparison of phosphorus recovery relative to co-extracted elements, phosphorus/metal (P/metal) extraction ratios were calculated (Table 3).

The P/metal ratios show that As was consistently extracted in greater amounts than P under all conditions, except in the second step of the sequential extraction where the ratio approached unity. This indicates that As co-extraction is unavoidable regardless of reagent choice. For the other metals, preferential P extraction was observed in the NaOH extractions and in the second step of the sequential EDTA–H_2_SO_4_ process. The sequential approach clearly improved selectivity compared with single-step acid extraction, demonstrating that staged dissolution can better balance P recovery and impurity control. Overall, these results highlight that alkaline extraction and sequential extraction strategies provide more favourable selectivity profiles than direct acid leaching. Based on the selectivity metrics, NaOH and the sequential EDTA–H_2_SO_4_ method offer the most promising pathways for selective phosphorus recovery.

#### 2.2.2. Impacts of Concentration

**Figure 2:**
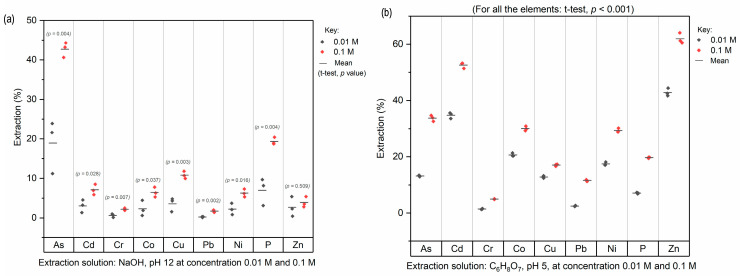

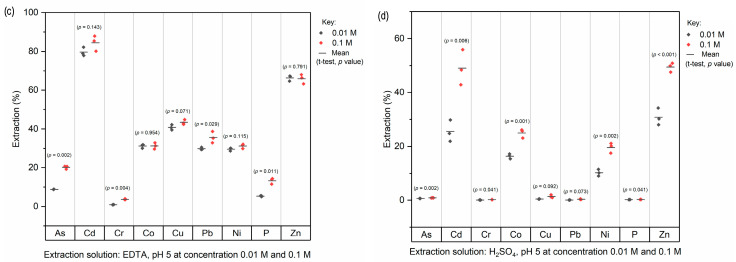
Impact of concentration variation for the extraction efficiencies of the reagents: (**a**) NaOH, (**b**) C_6_H_8_O_7_, (**c**) EDTA and (**d**) H_2_SO_4_ at concentrations of 0.01 M and 0.1 M, including *t*-test, *p*-values.

The results on the impact of concentration variation (0.01 M vs. 0.1 M) at similar pH of the reagent are shown in Figure 2.

It can be observed that generally, for all the elements, the reagents had higher extraction rates at 0.1 M as compared to 0.01 M at similar pH values (Figure 2). An independent *t*-test was carried out to compare the extraction rates at the different concentrations of the reagent (0.01 M and 0.1 M). This was done for the reagents at the same pH. For all the reagents, concentration variation resulted in a significant difference in the extraction efficiency for P (Figure 2).

For the metals, the EDTA concentration had no significant effect on the extraction of Cd, Co, Ni, and Zn. Variation in the concentration, however, had a significant effect on the extraction of As, Cr, and Pb (Figure 2c). With NaOH, except for Zn, there was a significant effect of the concentration on the extraction of the metals (Figure 2a). For citric acid, the concentration had a significant effect on the extraction rate of the reagent for all the elements (Figure 2b). For H_2_SO_4_, there was a significant difference in extraction rates for all the elements, with (Figure 2d). Across all the reagents, where there was a significant difference in extraction rates, higher extractions were observed at 0.1 M compared to 0.01 M.

These results are in line with other studies where an increase in the concentration resulted in increasing extraction rates [15,16,17,18,19,20]. This can be a result of an increase in the offered energy for breaking the metal chemical bonds promoted by an increase in the concentration [17]. An increase in the concentration results in an increase in the chances of collision between reactant particles in a specific time period since there will be more of the particles in the same volume. However, there reaches a point where with an increase in the concentration, the changes in extraction efficiencies were minimal.

Some studies have identified the most efficient concentrations as 0.02 M for EDTA [18] and 0.2 M for acids [17,18,20]. However, several factors come into play for the efficient extractant concentration, which include the initial concentrations of the materials and pH. Higher acid concentrations increase the rates of total phosphorus extraction [15]; however, economically very high concentrations will be more costly. In addition, this can result in surplus extractant ions in the solutions, which could otherwise be avoided, such as a surplus of sulphate ions where H_2_SO_4_ is the reagent. In the cases where there was no significant difference in extraction efficiencies with concentration variations, this could probably be because all the available fractions of the elements were already extracted.

#### 2.2.3. Impacts of pH for EDTA and H_2_SO_4_

**Figure 3:**
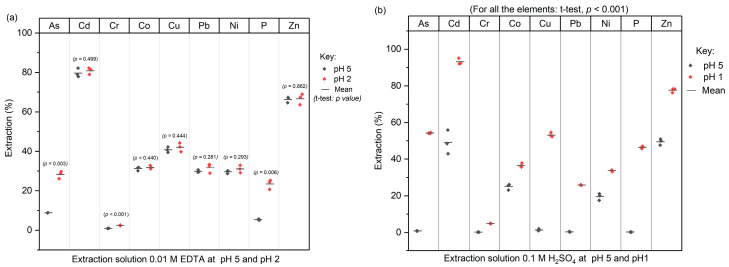
Impact of pH variation for the extraction P and the metals As, Cd, Co, Cr, Cu, Ni, Pb and Zn by (**a**) 0.01 M EDTA, pHs 5 and 2; and (**b**) 0.1 M H_2_SO_4_, pHs 5 and 1.

Figure 3 shows the impact of pH variations for the reagents 0.01 M EDTA and 0.01 M H_2_SO_4_ on their extraction rate.

There was a significant impact on the P extraction rate as a result of pH variation for both EDTA and H_2_SO_4_ (Figure 3a,b). For the metals, 0.01 M EDTA variation in pH had no significant effect on the extraction rate of most elements (Cd, Co, Cu, Pb, Ni, Zn) whilst it had a significant effect on the extraction rate of As and Cr (Figure 3a). In all the elements where there was a significant difference, there was a higher extraction at pH 2 than at pH 5. For H_2_SO_4_ also, variation in pH had a significant effect on the extraction rate of all the elements (Figure 3b). The 0.1 M H_2_SO_4_ had a higher extraction rate on the elements at pH 1 as compared to pH 5.

pH plays a pivotal role in the stabilisation and destabilisation of metal complexes. It can be found that the more acidic the leachate is, the more P and metals/metalloids can leach out. In this study, this was true for all elements for H_2_SO_4_, and for P, As and Cr for EDTA. A decrease in the pH of the solution often results in an increase in the extractability of most cationic metals, such as Pb, Cd, Zn, and Cu, due to a decrease in the adsorption of the cationic metals and an increase in the dissolution of the metal compounds [27]. Solubility of As oxyanions, on the other hand, increases when the solution pH increases. Low pH also promotes the adsorption of Cr(VI) anionic species [27].

In the cases where the pH had no significant effect on the extraction efficiencies of EDTA for Cd, Co, Cu, Pb, Ni, and Zn, this could be a result of similar stability constants at both pH values. EDTA has the following stability constants with the metal ions: Cr(II) 13.61, Cr(III) 23.4, Cd(II) 16.46, Co(II) 16.31, Co(III) 40.6, Cu(II) 18.8, Ni(II) 18.62, Pb(II) 18.04, and Zn(II) 16.5 [56,57]. The high stability constant values indicate that EDTA forms stable complexes with all the metal ions. In one study on metal extractions, EDTA portrayed the strong extraction of Pb up to pH 6 as a result of its high stability constant value [57].

#### 2.2.4. Impacts of the Sequential Two-Step Extraction

The purpose of having two steps for the extraction was to reduce the amount of metals in the final extraction after some had already been removed in the first step. The co-dissolution of metal with P in H_2_SO_4_ solutions has also been pointed out by other studies, such as [18].

**Figure 4:**
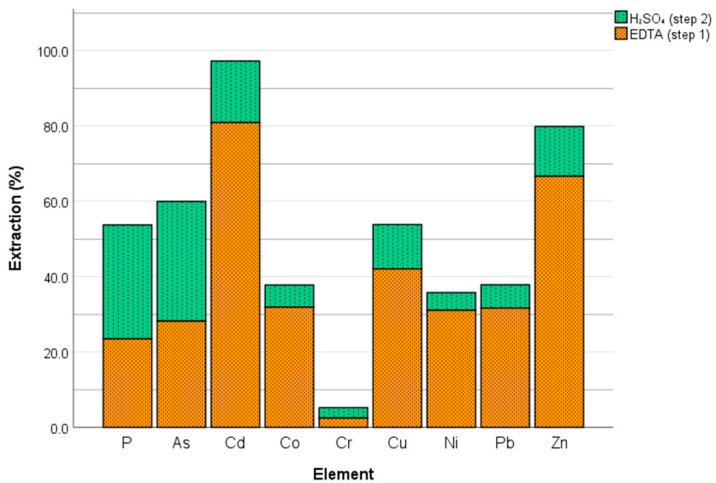
Impact of sequential two-step extraction, extraction efficiencies for P and the metals As, Cd, Co, Cr, Cu, Ni, Pb and Zn, where 0.01 M EDTA, pH 2 precedes 0.1 M H_2_SO_4_, pH 1 extraction.

From the two-step extraction procedure results, P was the second-lowest extracted element with EDTA, after Cr (Figure 4). This makes it a suitable pre-extractant as the goal is to recover more P in the final extraction. EDTA has also been reported to have a strong chelating ability for cationic trace metals and to cause limited destruction of the soil structure [27]. Element extractions with H_2_SO_4_ were in the order As > P > Cd > Zn > Cu > Pb > Co > Ni > Cr. With the exception of As, all the metals are below 18%, with Ni, Pb, Co and Cr below 10%. These values are much less than the metals otherwise extracted by the H_2_SO_4_ without prior treatment (4.9–93.1%). This is favourable environmentally and economically as less metal contamination would require fewer resources to be used for further purifying the recovered product.

For P, the cumulative extraction was 53.8%. About 46.3% of the P will still remain in the sediments and will still need to be managed to prevent nutrient contamination in the residual sediments. Except for Cr (5.2%), cumulative extraction of the elements was more than 35%, with Cd having the highest extraction (97.3%). This is an added advantage for the residual sediments as there are even fewer metal contaminants, as compared to having a single extraction with either EDTA or H_2_SO_4_. There is also high extraction of Cd, As, Cu, and Zn in the first step, which would otherwise raise further concerns on how to efficiently recover P for actual use.

**Figure A2:**
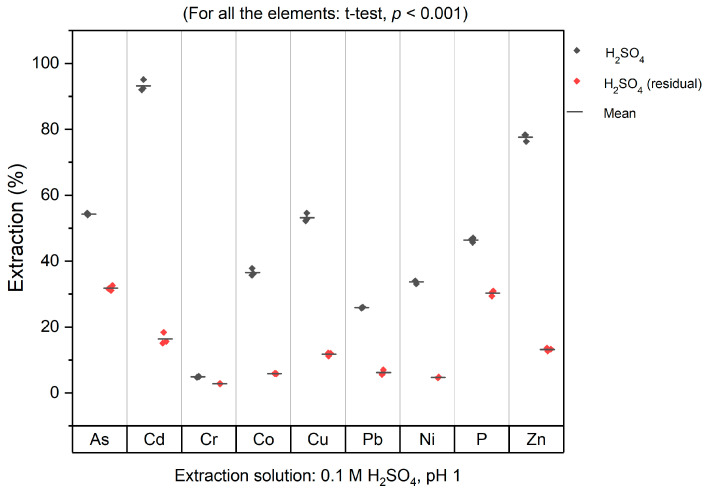
Impact of sequential two-step extraction, comparing the extraction efficiencies of 0.1 M H_2_SO_4_, pH 1, with *t*-test, *p* < 0.001 across all the elements: P and the metals As, Cd, Co, Cr, Cu, Ni, Pb and Zn. H_2_SO_4_ is a single extraction of 0.1 M H_2_SO_4_; and H_2_SO_4_ (residual) is 0.1 M H_2_SO_4_, in the sequential two-step extraction, where EDTA precedes 0.1 M H_2_SO_4_ extraction.

Comparing the extraction efficiencies of the H_2_SO_4_ at pH 1 (single extraction H_2_SO_4_ at pH 1 versus sequential two-step H_2_SO_4_ at pH 1), there was a significant difference in the extraction rates for all the elements. Further results for comparing the extraction efficiencies of the H_2_SO_4_ at pH 1 are shown in Appendix B Figure A2. Lower extractions were experienced with H_2_SO_4_ treatment after pre-extraction with EDTA. Although less P would be extracted as well (30.3 vs. 46.4%), a pre-treatment step with EDTA would be recommended to first reduce the metal pollutants in the sediments before extracting the P.

#### 2.2.5. Recommendation for Extraction

Several factors influence the extraction efficiencies, which include the reagent, concentration, extraction time, pH and liquid-to-solid ratio. One of the key drivers for this study was to find a suitable reagent for extracting P, which has a high P extraction rate and lower extraction of metals. The H_2_SO_4_ pH 1 solution had the highest extraction efficiency for P and also had the highest extraction efficiencies for the other elements as well, except for Cr and Pb (Figure 1). Hence, this would not be a suitable reagent on its own as there will be high metal contamination, adulterating P quality and requiring remediation of the extractant to avoid environmental contamination. P, As and Cd had their highest extraction in H_2_SO_4_ solutions together with P. The rest of the elements had their second-highest extractions in EDTA solutions. Cd had, however, its third-highest extraction from EDTA solutions. It can be concluded that EDTA would be an effective extractant for the contaminants, excluding As.

From the results, it has been observed that the metals are extracted at different rates in the different reagents. Other studies have reiterated that due to the different properties of elements, concurrent removal of cationic metals and anionic metalloids from soils by chelant/acid extraction is not effective [27,58]. Other researchers have suggested a combination of reducing agents (such as sodium oxalate, ascorbic acid and sodium dithionite) and chelating agents for the concurrent extraction of both anionic As and cationic trace metals from contaminated soils [58]. Hence, further studies can consider using the EDTA with a reducing agent to leach out more pollutants to get a cleaner P product during the subsequent recovery phase.

Both H_2_SO_4_ and EDTA have exhibited higher extraction efficiencies at low pH. It has been observed that the more metals/metalloids can be leached out, the more acidic the EDTA leachate, but when the pH < 1.7, EDTA reprecipitates [18]. Hence, the pH of 2 (used in the current study) would be recommended for EDTA. Increasing the EDTA concentration had no significant effect on the extraction of some of the elements (Cd, Co, Ni, Zn), although it had a significant effect on the other elements. From an economic perspective, the lower EDTA concentration of 0.01 M is recommended, and the concentration can be gradually increased. For H_2_SO_4_, the higher concentration of 0.1M is recommended.

The liquid-to-solid ratio is an important parameter, as there should be adequate contact between solid particles and the extracting solution. In one study, a decrease in the L/S ratio resulted in reduced contact efficiency and the extraction efficiency of P and all major cations [59]. The L/S ratio of 20 and extraction time of 2 h are good starting points as well and have been used in some of the previously mentioned studies (Table 1). On time, one study recommended the extraction time not be too long, as they observed a decrease in the P concentration and a general increase in the metal concentration in the liquid phase between 2 h and 1 week of extraction [19].

It should be taken into consideration, though, that EDTA is quite expensive, not readily biodegradable, and has the potential to remobilise trace metals in the environment, thus a cause of environmental concern [27,60]. It is, therefore, more resourceful to recycle and reuse the reagent instead of disposing of it in the environment. Some proposed methods for EDTA regeneration and the removal of the metals from the leaching solutions include the addition of chemical agents, nano-filtration, ion-exchange resin and electrochemical procedures [27]. One example of an electrochemical procedure is the use of sacrificial Al anodes, where the EDTA can be reused after separation from metals. EDTA environmental concern also applies to the residual sediments, which can be washed first before being disposed of in the environment.

Alternatively, NaOH can be used as an extractant. The reagent had P as its second-highest extracted element (Figure 3). Besides As, NaOH had the lowest extraction for the elements, with efficiencies of less than 10%. The P/metal extraction ratios also showed NaOH produced high selectivity, alongside the second step sequential extraction H_2_SO_4_. Although the potential P recovery will be low using NaOH, there is the least contamination with other elements, including Cd, which was highly extracted in the other reagents. Concern, however, will be on the As contamination. Cost-wise, it will also be cheaper to use NaOH as one reagent will be used instead of two reagents in the first alternative (H_2_SO_4_ and EDTA), which is an advantage. The cost of acquiring EDTA and subsequently recovering it from the environment is also avoided.

As sediment dredging will always be an ongoing process, recycling even a small portion of the P would be better than disposing of all of it in traditional ways with the sediments. After extraction, the next step will be recovering the P, for potential use in agriculture or industrial processes. Here, several methods can be used, which include wet chemical, thermochemical and electrochemical [31]. It is, however, important to take a step back and study the P speciation in the sediment to optimise the extraction processes, with known targeted P fractions for extraction and possible recoverable quantities. Environmental and economic feasibility studies are also crucial before implementing the potential P recovery processes at a larger scale. From previous studies on the sediments, the sediment disposal costs are approximately 0.07 Euro kg^−1^ at the local landfill, which is in the order of 1540 Euro per geobag of the sediments, and this can be compared to the cost of potential P recovery. A life cycle assessment for the process flow to recovery is one tool that can be employed as the next step, taking into account the costs for acquiring and treating extractants and sediment residues, as well as the avoided costs of exploiting virgin P resources and other environmental concerns.

## 3. Materials and Methods

### 3.1. Study Area

**Figure 5:**
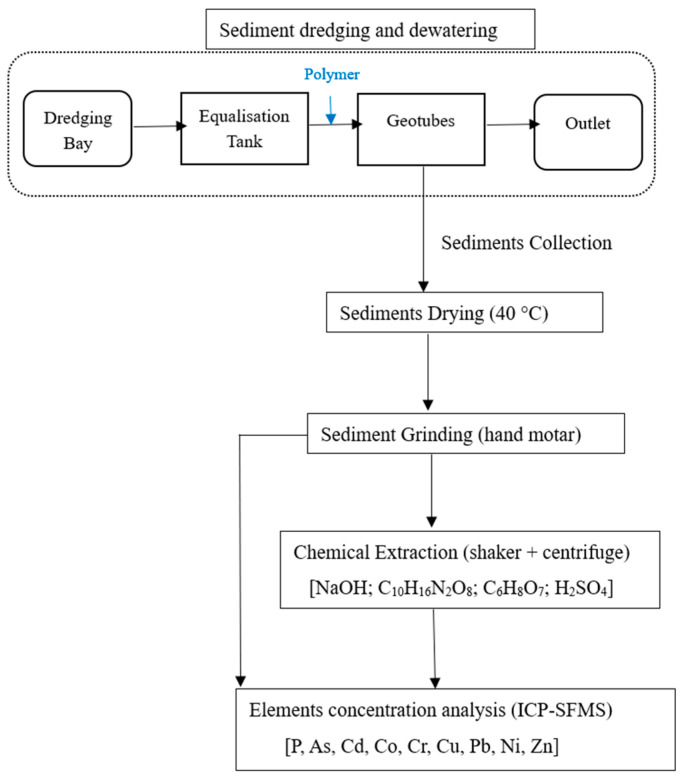
Summary of sediment preparation steps followed in the study. The arrows indicate the flow of sediment material, from dredging to element analysis.

The sediment used for the study was from Malmfjärden bay, Kalmar, Sweden. This is a shallow water body that is surrounded by residential and commercial areas. The bay is located just north of the town centre (56°66′ N, 16°36′ E), with minor connections to the Baltic Sea (Western Gotland basin). Figure 5 summarises the flow of sediment from the bay for element analysis.

### 3.2. Sediment Preparation

The LOVA: Mudster Eco Dredging project has a dewatering system for the dredged sediments, to increase the solid content of the sediments. The dredged material from the bay is received in an equalisation tank and passed to geotubes for dewatering. Dewatered sediments from the bay were used for this study. Sediments that were collected from the geobags and stored at 4 °C were dried at 40 °C and ground by hand. These were mixed with a previous batch of sediments from the same bay, which had already been dried, ground, and stored at room temperature in a closed plastic container. This was done to increase the representability of the sample and have sediments from different parts of the bay. The combined sediments were further dried together at 40 °C. Thereafter, the sediments were sieved together through 1.5 mm and stored in a closed plastic container. Since total element extraction was being carried out, we considered room-temperature storage in closed containers safe for the dried sediments.

### 3.3. Reagent Preparation

The chemicals used for the extractions included organic acids (citric acid), inorganic acids (sulphuric acid, nitric acid), sodium hydroxide and ethylenediaminetetraacetic acid (EDTA). These reagents were selected to try to cover a big range of agents, with acids (organic, inorganic), a base and a chelating agent. Two concentrations, 0.01 M and 0.1 M, were used for each reagent. These concentrations were selected based on the ranges used in previous studies such as [16,17,18,19]. Although some of these studies recommended 0.02 M and 0.2 M [17,18], for this study, it was decided to start at the lower concentrations since the material used in those studies (sludge ash) is different from the sediments. Solid reagents were weighed on an analytical balance. The solid reagents included EDTA (C_10_H_16_N_2_O_8_) (Acros Organics—Fair Lwan, NJ, USA), sodium hydroxide pellets (NaOH) (Acros Organics—Fair Lwan, NJ, USA) and citric acid (C_6_H_8_O_7_) (VWR Chemicals—Leuven, Belgium). Liquid reagents were pipetted using variable volume pipettes and a volumetric pipette. The liquid reagents included sulphuric acid (H_2_SO_4_) (Sigma-Aldrich—St. Louis, MO, USA) and nitric acid (HNO_3_) (Sigma-Aldrich—St. Louis, MO, USA) that were used for pH adjustment. For pH adjustments, 0.1 M HNO_3_ and 0.1 M NaOH were used. The water used was deionised water. The solutions were prepared in pre-acid-washed beakers, and mixing was done using magnetic stirrers. The prepared solutions were then placed in acid-washed glass bottles with lids and labelled accordingly.

### 3.4. Extraction Sample Preparation

Equal liquid-to-solid (L/S) ratios (1:20) were used for all samples, based on previous studies such as [18,19]. To 2.5 g sediment, 50 mL of reagents was added. The samples were done in triplicate for each experiment. For each reagent concentration, there was a blank. The pH was adjusted accordingly with NaOH or HNO_3_. All pH measurements in this study were done using a pH metre (HQD field case, Hach Lange—Düsseldorf, Germany). One test involved treatment with two steps (Table 2). The first step involved extraction with EDTA. After removing the extractant, sulphuric acid was then added to the residual sediment for the second extraction step. This test was done based on a recommendation from a previous study [18], to reduce the concentration of metals in the final extract solution due to the removal by EDTA in the first step. Table 4 shows the solutions used during the experiments.

### 3.5. Extraction Process

Prepared samples were placed on a test tube rack and shaken on a shaker (GFL 3015, GFL Gesellschaft für Labortechnik mbH—Burgwedel, Germany) for 2 h at 300 rpm. The extraction time was based on previous studies [17,18,19,20]. Thereafter, the samples were centrifuged (Beckman Avanti J-25, Beckman Instruments Inc.—Fullerton, CA, USA) at 4000 rpm for 30 min and filtered through 0.45 μm membrane filters (Frisenette ApS—Knebel, Denmark).

### 3.6. Element Concentration Analysis

Element concentration analyses were done for the filtered samples, together with dried and ground sediment samples (duplicate). The analyses were performed by ALS Scandinavia AB, Luleå, Sweden, an ISO/IEC 17025:2017 [61] accredited testing laboratory (SWEDAC; accreditation no. 2030).

The elemental concentration analysis included for phosphorus (P), cadmium (Cd), lead (Pb), arsenic (As), cobalt (Co), chromium (Cr), copper (Cu), nickel (Ni) and zinc (Zn). For the analysis of the filtered extraction samples, inductively coupled plasma sector field mass spectrometry (ICP-SFMS) was used according to SS-EN ISO 17294-2:2016 [62] and US EPA Method 200.8:1994 [63]. For the analysis of the element concentrations in the sediment samples, an ICP -SFMS was employed according to SS-EN ISO 17294-2:2016 and US EPA Method 200.8:1994. Prior to analysis, the sample was digested in 7 M nitric acid in a hot block according to SE-SOP-0021.

As part of the accredited quality system, ALS applies a complete QA/QC procedure including method blanks, laboratory control samples, matrix spikes, duplicate samples, and certified reference materials (CRMs) appropriate for trace metal analysis in environmental matrices. Instrument calibration was performed using multi-point external standards with internal standard correction. Method detection limits (MDLs) and reporting limits (RLs) followed ALS standard protocols. Analytical recoveries for CRMs were within the acceptance limit, and duplicate analyses showed precision within the required limits. These QA/QC procedures ensure the accuracy and reliability of the trace metal concentrations reported in this study.

### 3.7. Data Analysis

To find the extraction efficiencies of the reagents, the extraction rates for the elements were calculated relative to the total element content in the sediment (Equation (1)). During the sequential two-step extraction experiment, for the second step extraction with H_2_SO_4_, the extraction rates were also based on the total element content of the sediment after pre-step EDTA extraction.

(1) $$Eff=\frac{{Mass}_{f}}{{Mass}_{i}}\%,$$

where *Eff* is the extraction efficiency, *Mass_i_* is the initial mass of the element in the sediment sample, and *Mass_f_* is the extracted mass of the element from the sediment sample.

For direct comparison of phosphorus extraction relative to co-extracted elements, phosphorus/metal (P/metal) extraction ratios were calculated (Equation (2)). P/metal extraction ratios < 1 indicated poor selectivity, P/metal extraction ratios = 1 indicated no selectivity and P/metal extraction ratios > 1 indicated good selectivity.

(2) $$P_{Metal\;extraction\;ratio}=\frac{Phosphorus\;extraction}{Metal\;extraction}$$

Analysis of Variance (ANOVA) was used for identifying significant differences in the extraction rates of the reagents, with *p*-values < 0.05 considered a significant difference. Homogeneity of variance was tested (Levene statistic). Post hoc tests were run using Tukey HSD and Games–Howell for where ANOVA presented a significant difference. For comparison of the concentration and pH, an independent *t*-test was used with *p*-values < 0.05 considered a significant difference. Levene’s test for equality of variances was considered for the two-tailed test. Concentration comparisons were done for the reagent at the same pH and pH comparisons were also done for the reagent at the same concentration. The statistical analyses were calculated using IBM SPSS Statistics Version 27.

## 4. Conclusions

The aim of the study was to extract phosphorus from dredged sediments from Malmfjärden bay through chemical extraction and also to determine the best conditions to extract phosphorous from the studied sediments with minimal contamination with metals. Since this was a preliminary study, the results constitute an initial extraction assay but not the establishment of an effective operational framework for phosphorus recovery. In all the experimental conditions, some P was extracted with efficiencies of 0.2% to 46.4%. H_2_SO_4_ had both the lowest and highest P extraction efficiencies, an indicator of how several parameters, such as the time, L/S ratio, pH and concentration (and not just the reagent), influence the extraction rate. An increase in both the acidity and concentration increased the rate of extraction. Co-dissolution of metals with the P is inevitable, and from the preliminary study, there could be potential in pre-treating the sediments by extracting with EDTA first, before using H_2_SO_4_. Alternatively, there is also potential with NaOH, as it has the lowest metal extraction efficiencies. The P/metal extraction ratios also showed NaOH produced high selectivity, alongside second step sequential extraction H_2_SO_4._ Further research on the environmental impacts of residual sediments and extractants must, however, be considered. Research on the P speciation in the sediments should also be considered, for a better understanding of the P forms in the sediments, which will aid in optimising the extraction procedures.

From the current study and previous studies with Malmfjärden sediments, the sediments have potential as a resource and not a waste. The sediments can be safely used in less sensitive land use areas, and if they are to be used in sensitive land use areas, prior remediation can be done to tone down the concentrations of metals such as Cd, Pb, Zn and As. There is potential for P extraction from sediments as one way of recovering the finite element, which would otherwise be normally lost as waste with the sediments. Economic feasibility, however, must be considered. This includes further research on recovering the P from the extractant with higher P purity and less contamination from metals/metalloids.

## Figures and Tables

**Table 1:** Summary of some studies where P has been extracted from solid material and the extraction conditions.

Materials and Chemicals	Chemicals	Concentration	Time	pH	L to S	References
Pig manure solids	Citric and hydrochloric acids	2.5, 5, 10, 20, 40, and 80 mmol L^−1^	1 h	Citric acid 2.5–5.4 HCl 0.2–5.9	1:25 *w*/*v* ratio	[15]
Soil	Citric acid, EDTA and water	0.01 M	1 h	5	10 g sample: 30 mL solution	[16]
Sewage sludge ash	Hydrochloric acid and NaOH	0.01–0.8 mol/L	2 h		25–150 mL/g	[17]
Incinerated sewage sludge ash	Sulphuric acid, nitric acid, oxalic acid, citric acid, EDTMP and EDTA	0.1, 0.2, 0.5 mol/L for other acids 0.01, 0.02, 0.05 mol/L chelatins	2 h	0.85–2.03 other acids 1.32–4.84 chelatins		[18]
	H_2_SO_4_ and EDTA	0.1, 0.2, 0.3, 0.4, 0.5, 0.7, 1 mol/L H_2_SO_4_ 0.01, 0.02, 0.03, 0.04, 0.05 EDTA	(10, 30) mins, (1, 2, 4, 6 and 24) h	1, 2, 3, 4, 5 H_2_SO_4_ 1.7, 2.9, 7, 9.3 and 12.6 EDTA	10, 15, 20, 25, 30, 40 and 50	
Incinerated sewage sludge ash	HNO_3_ H_2_SO_4_	0.01 M to 1.5 M 0.19 M 0.08 and 0.28 M	1 week 2 h or 1 week 2 h	pH measured (1–10)	2.5, 5, 10 and 20	[19]
Municipal solid waste incineration fly ash	HCl NaOH (precipitate)	1, 2 and 3 M 1.5 and 2.5 M 1 M 1 M	2, 4 and 24 h 2 h only 1 h 5 h	3, 4 3 4	5	[20]
	HCl NaOH	1 M 1 M	2 h 4 h	4 11–13		

**Table 2:** Concentration of elements in the sediments in mg kg^−1^ DW (mean ± SD, *n* = 2) in comparison to the Swedish guidelines for sensitive land use and less sensitive land use [42].

Element	Concentration (mg kg^−1^ DW)	Swedish Guidelines [42]	
		KM Limit	MKM Limit
Phosphorus (P)	1080 ± 0	-	-
Arsenic (As)	9.44 ± 0.17	10	25
Cadmium (Cd)	2.17 ± 0.04	0.7	2.5
Chromium (Cr)	31.5 ± 2.1	80	150
Cobalt (Co)	9.96 ± 0.06	15	35
Copper (Cu)	67.4 ± 0.9	80	200
Lead (Pb)	83.8 ± 0.5	50	400
Nickel (Ni)	27.9 ± 0.99	40	120
Zinc (Zn)	258 ± 7	250	500

Key: DW—dry weight, KM limit—(Känslig markanvändning)—sensitive land use limit, MKM limit—(Mindre känslig markanvändning)—less sensitive land use limit, 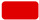 surpass the KM limit, 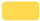 below but close to the KM limit.

**Table 3:** P/metal extraction ratios from the extraction solutions (with ratios ≤ 1 highlighted in orange).

Concentration		0.01 M	0.1 M	0.01 M	0.1 M	0.01 M	0.1 M	0.01 M	0.1 M	0.1 M	0.01 M	0.1 M
pH		5	5	12	12	5	5	5	5	1	2	1
	Reagent	EDTA	EDTA	NaOH	NaOH	C_6_H_8_O_7_	C_6_H_8_O_7_	H_2_SO_4_	H_2_SO_4_	H_2_SO_4_	Sequential Two-Step Extraction	
P/Metal Extraction											EDTA	H_2_SO_4_
P/As		0.6	0.7	0.4	0.5	0.5	0.6	0.3	0.3	0.9	0.8	1.0
P/Cd		0.1	0.2	2.3	2.7	0.2	0.4	0.0	0.0	0.5	0.3	1.8
P/Cr		5.8	3.7	11.1	8.8	4.9	3.9	2.9	1.3	9.5	9.6	10.8
P/Co		0.2	0.4	3.0	3.0	0.3	0.7	0.0	0.0	1.3	0.7	5.2
P/Cu		0.1	0.3	2.1	1.8	0.6	1.2	0.5	0.2	0.9	0.6	2.6
P/Pb		0.2	0.4	29.1	11.2	2.9	1.7	2.9	0.7	1.8	0.7	4.9
P/Ni		0.2	0.4	3.2	3.1	0.4	0.7	0.0	0.0	1.4	0.8	6.4
P/Zn		0.1	0.2	2.6	5.0	0.2	0.3	0.0	0.0	0.6	0.4	2.3

**Table 4:** Extraction sample preparation, with the concentrations and pH of solutions.

Experiment	Solution Reagent	Concentration of Reagent	pH of Solution
1	NaOH	0.01 M	12
2	NaOH	0.1 M	12
3	Citric acid	0.01 M	5
4	Citric acid	0.1 M	5
5	EDTA	0.01 M	5
6	EDTA	0.1 M	5
7	Sulphuric acid	0.01 M	5
8	Sulphuric acid	0.1 M	5
9	Sulphuric acid	0.1 M	1
10-a	EDTA	0.01 M	2
10-b	Sulphuric acid (Extraction of residue from 10a)	0.1 M	1

## Data Availability

The data for this study will be shared upon reasonable request to the authors.

## Abbreviations

The following abbreviations are used in this manuscript:

P	Phosphorus
C_6_H_8_O_7_	Citric acid
H_2_SO_4_	Sulphuric acid
NaOH	Sodium hydroxide
EDTA	Ethylenediaminetetraacetic acid
KM	(Känslig markanvändning)—sensitive land use limit
MKM	(Mindre känslig markanvändning)—less sensitive land use limit
LOVA	(Lokala vattenvårdsprojekt)—Local Water Conservation Projects
As	Arsenic
Cd	Cadmium
Co	Cobalt
Cr	Chromium
Cu	Copper
Ni	Nickel
Pb	Lead
Zn	Zinc
L/S	Liquid-to-solid ratio
